# Editorial: The Role of Dietary Interventions in The Regulation of Host-Microbe Interactions

**DOI:** 10.3389/fcimb.2022.849508

**Published:** 2022-02-03

**Authors:** Shengjie Li, Xiumiao Zhao, Tingtao Chen

**Affiliations:** ^1^ The Institute of Translational Medicine, Nanchang University, Nanchang, China; ^2^ Queen Mary School, Nanchang University, Nanchang, China

**Keywords:** dietary interventions, gut microbiota homeostasis, dysbiosis, probiotics/prebiotics, *Lactobacillus*, immune response, host health

The gut microbiota, a dynamic ecosystem composed of trillions of microbes in the gastrointestinal tract, has been demonstrated to be involved in the maintenance of human well-being. Mounting evidence has indicated that the diet can be regarded as one of the most effective modulators to the composition and functionality of gut microbiota. While in turn, the gut microbiota profoundly influences the digestion of dietary components, the process of nutrient absorption, and energy storage. This symbiotic relationship between diet and gut microbiota further affects the host physiological functions, suggesting that any perturbation of it may cause varying degrees of discomfort in hosts. Therefore, the gut microbiota could serve as a bridge in the close interaction between dietary interventions and host health. Collectively, these suggestions indicate that targeting the diet could be a potential therapeutic strategy for human diseases *via* modulating the gut microbiota.

Till now, probiotics/prebiotics have been generally proposed as dietary intervention strategies or adjuvant treatments for managing multifarious diseases, such as cancers, gastrointestinal disorders, enteric infections, immune-mediated allergic and autoimmune diseases, and psychiatric disorders. Although various hypotheses have demonstrated that probiotics/prebiotics supplementation plays health-promoting roles *via* targeting the gut microbiota and/or regulating the systemic immune and metabolic responses, the precise and common molecular mechanisms of these beneficial roles of dietary factors on the host health still need to be explored deeply.

This current Research Topic brings twenty-three references together summarizing the recent developments pertaining to the characterization of probiotics (mainly *Lactobacillus*, *Bifidobacterium*, and *Saccharomyces*) isolated from different niches, the potential relevance of intestinal dysbiosis in the progression of host diseases, and the underlying mechanisms of dietary supplementations as novel strategies for the prevention and treatment of diseases by modulating the gut microbiota.

The exploration of the influence of dietary/probiotics interventions in cancer treatment and adjuvant therapy and the associations between the gut dysbiosis and cancers, have been the focus of recent research. A randomized clinical trial performed by Xia et al. suggested a modified probiotic cocktail that contains *L. plantarum* MH-301, *B. animalis* subsp. *Lactis* LPL-RH, *L. rhamnosus* LGG-18, and *L. acidophilus*, could attenuate the severity of oral mucositis induced by chemo-radiotherapy in nasopharyngeal carcinoma. It outlined that the efficacy of this cocktail was associated with reframing the gut dysbiosis, reducing cell apoptosis, preventing inflammation, and enhancing the host immune response. Remarkably, the pathogenesis of colorectal cancer (CRC) from the perspective of the gut microbiota has been comprehensively reviewed by Cheng et al. The review discussed the microbiota-derived inflammation, bacterial dysbiosis (metabolites and pathogens), virulence factors, oxidative stress, genotoxins, and biofilm, which all related with the progression of CRC. It also suggested that considering the intestinal microbiota as a therapeutic strategy would display higher clinical value on the prevention and treatment of CRC in the future.

The severity of clinical manifestations of enteric infection ranges from mild diarrhea, abdominal pain, blood in stool, maldigestion to septic shock, and even death. And probiotics could be used as a promising antibiotics replacement therapy to alleviate these symptoms. As Gaisawat et al. described, several probiotics, such as *L. rhamnousus* R0011 and *S. boulardii* CNCM I-1079, were able to modulate *Clostridioides difficile*-infected (CDI) inflammatory responses in a novel systematic testing approach. They demonstrated that CDI microbiota-derived fecal water adversely caused cytotoxic damages to colonic cells by increasing the production of various inflammatory markers (e.g., TNFSRF8, IL-32, and MIF), which would be counteracted by probiotics supplementation. Besides, Quilodrán-Vega et al. isolated potential probiotic/immunobiotic *Lactobacillus* from llama milk and assessed their adaptability in the gastrointestinal environments, antibacterial properties against pathogens, adhesive and immunomodulatory activities *in vitro*. Among these, the *Ligilactobacillus salivarius* TUCO-L2 showed enhanced beneficial potentials with higher resistance to extreme conditions and more potent adhesion effects to mucins and epithelial cells. Moreover, this strain has been reported to modulate the TLR-4 mediated innate immune responses and improve the protective effects against *Salmonella* infection *in vivo*, which indicated that it could be used as a probiotic or immunobiotic strain to combat infections in mammals. Another study performed by Zhou et al. illustrated the underlying mechanisms of *Lactobacillus* in fighting against enteric *Salmonella* infection using *Caenorhabditis elegant* model. It suggested that *L. zeae* LB1 could regulate the p38 MAPK pathway, the production of antimicrobial peptides, and the expression of defense molecules, to combat *Salmonella* infection.

Increasing evidence has illustrated that probiotic/prebiotics play a variety of promoting roles in the management of gastrointestinal disorders due to their possible immunomodulatory and gut microbiota-sustaining activities. For instance, a study presented by Li et al. reported that *Clostridium butyricum* RH2 could restore mucosal barrier function and ameliorate ceftriaxone-induced dysbacteriosis *via* enhancing T cells immune response and modulating the changed gut microbiota. In addition, Xu et al. achieved similar intervention effects of DHA algal oil (a promising dietary nutrition possessing anti-inflammatory function in various clinical trials) on colitis mice, highlighting the potential roles of algal oil in treating colitis and other intestinal dysfunction. Liu Y et al. also proved that *L. plantarum* CCFM8610 could protect against Cadmium (Cd) induced intestinal dysmotility in mice by reducing Cd levels in the body, restraining the cellular stress response, and recovering neurotransmitter levels.

Recently, shreds of evidence have suggested that the development of immune-mediated allergic diseases is associated with the host-microbiome, especially the perturbation of intestinal microbiota that may contribute to their progression which could be alleviated by dietary probiotic supplements. In their paper, Fang et al. reviewed the changes of gut microbial in patients suffering from atopic dermatitis (AD) and discussed the underlying mechanisms of probiotics on ameliorating AD’s clinical manifestation. They also highlighted that targeting the gut microbiota is an effective strategy for preventing and treating AD. Thus, probiotic supplements might be an alternative for the alleviation of AD *via* the “gut-skin axis”. Moreover, Xie et al. assessed the effects of different dietary patterns on *L. paracasei*-modulated allergic asthma (AA). They discovered that the consumption of high-fiber assists *L. paracasei* in alleviating allergic symptoms and airway inflammation by regulating gut microbiota and improving immune response. They emphasized that a high fiber diet benefits probiotic medication on AA, which could be considered as an add-on therapy for gut microbiota-associated disorders.

Intestinal dysbiosis, mainly manifesting as bacterial diversity and function alterations, may also cause autoimmune diseases. As described by Cayres et al., patients with Hashimoto thyroiditis (HT), a commonest organ-specific autoimmune disorder, showed the symptoms of gut microbiota alterations and intestinal permeability, indicating that the gut dysbiosis associated closely with diet habits might contribute to the development and progression of HT. Furthermore, they discussed the potential roles of diet in modulating HT’s performances *via* the diet-microbiota-immune system axis. In addition, probiotics could be also used as adjuvant therapy to treat autoimmune diseases. In this Research Topic, Fan et al. found that *L. casei* CCFM1074 with abundant carbohydrate metabolism genes, could relieve arthritis in the rat model by modulating gut microbiota and plasma metabolites, rebalancing the immune responses (especially the ratio of Treg/Th17 cells) and down-regulating the pro-inflammatory cytokines, which provided a thorough insight into the probiotics in curing arthritis. A study presented by Emu et al. in the livestock industry also suggested that probiotics (*S. cerevisiae* and mannan oligosaccharides) supplementation can improve the growth and immunity of black fattening goats by positively promoting the growth of beneficial gut microbes, such as *Akkermansia muciniphila* and *Ruminococcus flavefaciens*.

Chronic metabolic disorders, such as obesity, diabetes, and metabolic dysfunction-associated fatty liver disease, have also been verified to associate closely with gut dysbiosis. As presented by Du et al., resveratrol (RSV) reduced liver steatosis and insulin resistance in non-alcoholic fatty liver disease (NAFLD) by directly modulating the gut microbiota *in vivo*, which strengthened the molecular mechanisms of RSV as a therapeutic drug for NAFLD. Another study published in this Research Topic by Higgins et al. illustrated that disruptions of metabolic phenotype and gut microbiota were apparent in the mouse model with western diet-induced obesity. Apart from the common bacteriome, the bacteriophage communities also had a dynamic relationship with their bacterial hosts during the progression of obesity. Furthermore, this study highlighted the novel correlations between diet-induced metabolic changes and intestinal microbiome constituents, which helps explain the pathophysiology of obesity.

However, inconsistencies of findings describing the differences of gut microbiota within and across individual studies raise attention to identifying the distinct features of gut microbiota in these disorders. Therefore, Que et al. performed a meta-analysis to explore the differences in stool microbial profiles between individuals with type 2 diabetes mellitus (T2DM) and controls by using seven previous studies’ sequencing datasets. They suggested that the overall microbial communities but not alpha diversity are significantly different in T2DM patients. Several OTUs or bacterial genera related to *Dorea*, *Clostridium*, and *Lactobacillus* were identified as the microbial signatures for predicting diabetes. Moreover, they discussed the correlations between T2DM-associated genera and probiotics, suggesting that the adjustment of gut microecology by probiotics is a new strategy for preventing and treating T2DM.

In pediatrics, the colonization of the gut microbiome in early life has been demonstrated to be vital for infant development, which could be highly influenced by feeding patterns and maternal dietary habits. In this Research Topic, Zhu et al. performed a pilot randomized clinical trial to compare the effects of two infant formulas containing OPO (1, 3-olein-2-palmitin) and other prebiotics (fructo- and galacto-oligosaccharides) separately on the fecal microbial composition in enrolled infants, and achieved similar feeding outcomes to those with breastfeeding. Their findings suggested that giving formula with prebiotics and OPO is beneficial to shape the function and structure of infant gut microbiota, indicating that these additives might help to modulate intestinal microbiota and promote the development of infants and children. Another study performed by Ye et al. suggested the neonatal milk fat globule membrane (MFGM, the sole source of phospholipids in breast milk) supplementation during breastfeeding could counteract the maternal high-fat diet (HFD) induced metabolic disorders in adult offspring by modulating their gut microbial structure. Intriguingly, this article also suggested that the shifts of the gut microbiome and its related metabolism showed sex dimorphism in the offspring.

As a mediator between the diet and the host, the gut microflora can generate metabolites, such as short-chain fatty acids (SCFAs), by fermenting dietary components to preserve gut homeostasis. Increasing evidence illustrates that SCFAs could cross the blood-brain barrier (BBB) to perform their health-promoting effects in multiple brain disorders. As Liu et al. presented, SCFAs administration improved behavioral impairment and exerted neuroprotective impacts against sepsis-associated encephalopathy (SAE) in mice. Significantly, the pretreatment of SCFAs could ameliorate neuronal degeneration, repair the disrupted BBB by increasing the expression of ZO-1 and Occludin, reducing the neuronal inflammation by the overactivation of microglia cells, and decreasing pro-inflammatory cytokines (IL-1β and IL-6). Moreover, SCFAs blocked the NF-κB and JNK pathways by inhibiting the phosphorylation of NF-κB p65 and JNK to suppress the neuroinflammation of SAE.

Generally, *Lactobacillus* are considered as probiotics in host niches. However, Zhang F et al. contradicted this inherent view by summarizing recent findings on the adverse effects of *Lactobacillus* in gynecology. They suggested that the adherent abilities of vaginal *Lactobacillus* play a bidirectional role in regulating the female reproductive health. On the one hand, opportune adherent effects of vaginal *Lactobacillus* are crucial for maintaining the homeostasis of the intrinsic vaginal microbiota. On the other hand, these adherent effects may impair the agglutination and immobilization of the ejaculated sperm, resulting in an unexplained infertility. But the shifts of vaginal microbiota from the *Lactobacillus* dominant state to a polymicrobial microbiota one with an increased diversity of anaerobic bacteria, often cause bacterial vaginosis (BV) that is associated with various women’s gynecological conditions, e.g., adverse pregnancy and infertility. Many efforts are trying to treat BV due to the high incidence of recurrence after antibiotics therapy. Zhang Q-Q et al. reported that maltose gel might be an alternative prebiotic agent for treating BV *via* promoting vaginal microbiota from the BV-related bacteria dominant microbiota to the *Lactobacillus* dominant one in *Rhesus macaque*.

Although probiotic possesses active roles in alleviating various conditions, the reduced viability of probiotics after administration from mouth to the functional sites is a great trouble for consumers and producers. In their review, Han et al. summarized that many harsh factors caused the diminished benefits of probiotics during their journey in the gastrointestinal tract, including low pH value in the stomach, high concentration of bile salts and digestive enzymes in the small intestine, and colonization resistance in the colon. Notably, they discussed the underlying mucoadhesion mechanisms and several adhesive proteins involved in the colonization of probiotics, explaining how probiotics communicate with commensal microorganisms and why some strains can successfully introduce into the gut microbiota. The genomic variations of a kind of *Lactobacillus* have strong impacts on its phenotypes, which brings much concern to the exploration and application of a specific kind of probiotic genus from different niches. Taking *L. plantarum* as an example, Pan et al. found that niche-specific genomic variations have been identified in these strains. Furthermore, they discussed how these variations affect their metabolic abilities and interactions with host intestinal flora, providing a novel guideline for probiotic exploitation and application in the future.

Overall, this Research Topic emphasizes that the diet, microbiome, and their interplay with the host are essential factors in maintaining gut homeostasis, or are potential causes of diseases associated with gut dysbiosis. In particular, dietary supplementations with probiotics/prebiotics play a vital role in modulating intestinal functions and enhancing host systemic immunity. However, the current Research Topic has a few limitations. The accepted papers span across the animal and human sphere and cover various diseases, making this Research Topic might be a bit broad and disorder. And there seems to be few deep discussions concerning “mechanism” in these current literatures, and even if there are, most of the mechanisms look usually scattered. Furthermore, the functional studies of specific dietary supplements and probiotic strains have verified that the natural individual differences contribute greatly to the distinctiveness of gut microbial community between healthy and disease status. Therefore, multi-omics techniques, e.g., cultivating omics, metabolomics, metagenomics, and other omics, combined with high-throughput analysis are desperately needed to identify the exact cross-links between diet, host-microbes, and host beings.

## Author Contributions

SL, XZ, and TC wrote and revised this article. All authors made a substantial, direct, and intellectual contribution to this work and approved it for publication.

## Funding

This work is supported by grants from the National Natural Science Foundation of China (Grant no. 82060638), and Double thousand plan of Jiangxi Province (high end Talents Project of scientific and technological innovation).

## Conflict of Interest

The authors declare that the research was conducted in the absence of any commercial or financial relationships that could be construed as a potential conflict of interest.

## Publisher’s Note

All claims expressed in this article are solely those of the authors and do not necessarily represent those of their affiliated organizations, or those of the publisher, the editors and the reviewers. Any product that may be evaluated in this article, or claim that may be made by its manufacturer, is not guaranteed or endorsed by the publisher.

